# Insights into the natural history of metachromatic leukodystrophy from interviews with caregivers

**DOI:** 10.1186/s13023-019-1060-2

**Published:** 2019-04-29

**Authors:** Magdalena Harrington, Diane Whalley, James Twiss, Rebecca Rushton, Susan Martin, Lynn Huynh, Hongbo Yang

**Affiliations:** 1 Shire, a member of the Takeda group of companies, Lexington, MA USA; 2 Present address: Pfizer, 610 Main St, Cambridge, MA 02139 USA; 3 RTI Health Solutions, Manchester, UK; 4 RTI Health Solutions, Ann Arbor, MI USA; 5 Analysis Group, Inc., Boston, MA USA

**Keywords:** Metachromatic leukodystrophy, MLD, Lysosomal storage disease, Caregiver, Qualitative research, Natural history

## Abstract

**Background and methods:**

Metachromatic leukodystrophy (MLD) is a rare, autosomal recessive lysosomal storage disease caused by deficient activity of arylsulfatase A. Neurological involvement results in severe disability and premature death, but understanding of the natural history of the disease remains limited. In this study, 32 caregivers of patients with MLD in the USA (16 with late-infantile MLD; 16 with juvenile MLD) were interviewed about their experiences of the disease. Qualitative analysis of the interview transcripts was performed to gain insights into symptom onset, the diagnostic process and disease progression, with a focus on the differences between late-infantile and juvenile MLD.

**Results:**

The mean ages of patients at interview were 7.6 years and 20.7 years for individuals with late-infantile and juvenile MLD, respectively. Patients with late-infantile MLD had a mean age of 1.5 years at symptom onset and 2.6 years at diagnosis. The most common initial symptoms in this group related to problems with gross motor function (12/16 patients); 11 patients never learned to walk independently. For patients with juvenile MLD, the mean ages at symptom onset and diagnosis were 8.7 years and 11.6 years, respectively. Cognitive or social/behavioural problems were the most common first reported symptoms in this group (9/16 and 7/16 patients, respectively); these were generally followed by deterioration in motor function. The rate of functional decline was more rapid in patients with late-infantile MLD than those with juvenile MLD; the mean time from first symptom to first functional loss was 1 year versus 6.1 years, respectively. Nine patients with juvenile MLD and three with late-infantile MLD had undergone a haematopoietic stem cell transplant; outcomes following transplant were variable.

**Conclusions:**

Our data highlight clear overall differences in symptom profiles and disease progression between late-infantile and juvenile MLD, but also indicate some degree of interindividual variability within each subtype. These findings are broadly consistent with previously published descriptions of MLD and enhance our knowledge of the natural history of the disease, which ultimately should help to improve patient care and aid assessments of the effectiveness of disease-related interventions in the future.

**Electronic supplementary material:**

The online version of this article (10.1186/s13023-019-1060-2) contains supplementary material, which is available to authorized users.

## Background

Metachromatic leukodystrophy (MLD; OMIM 250100) is a rare, autosomal recessive lysosomal storage disease caused by a functional deficiency of the lysosomal enzyme arylsulfatase A (ASA or ARSA) [1, 2]. ASA deficiency leads to the accumulation of sulfatides in the central and peripheral nervous systems, which is associated with demyelination and consequent neurodegeneration [1, 2]. Patients with MLD generally experience progressive loss of gross and fine motor functions and a severe decline in cognitive function, ultimately leading to premature death [1–4].

MLD is commonly classified into three clinical subtypes depending on the age of onset: late-infantile, juvenile or adult (< 30 months, 2.5–16 years and > 16 years, respectively) [5]. The late-infantile form is generally associated with rapid and severe functional decline, while patients with the juvenile and adult forms tend to experience a slower rate of disease progression [2–4, 6, 7]. Impairments in gross motor function, such as a failure to develop independent walking, are frequently reported first for patients with late-infantile MLD [3, 4, 8]. For patients with the later-onset forms, however, cognitive and behavioural signs and symptoms are often the earliest indicators of disease, followed by a more protracted decline in motor function [3, 4, 8]. While some correlations have been observed between specific pathogenic mutations and disease severity, the clinical course of MLD has been reported to vary significantly even among siblings, suggesting that unknown factors may also influence disease phenotype [1, 9, 10].

There is currently no disease-specific curative treatment for MLD, and management is therefore typically palliative [2, 5]. Haematopoietic stem cell transplantation (HSCT) has been performed in some patients with MLD, and has been reported to stabilize or delay disease progression in certain patients who received the transplant at a pre-symptomatic or very early symptomatic stage [5, 11–15]. However, this procedure is high-risk, has generally shown little benefit in patients with late-infantile MLD, and has variable outcomes even in patients with later-onset disease [5, 11–15]. Gene therapy [16–19] and intrathecal enzyme replacement therapy [20] are also under investigation as potential therapeutic approaches for MLD; however, further research is required to evaluate their long-term safety and efficacy.

To improve patient care and to be able to evaluate the effectiveness of potential therapies, it is important to increase our understanding of the natural history of MLD and the experiences of patients. Caregivers of patients with MLD represent a valuable source of information in this respect, because their close, daily contact with patients enables them to provide detailed accounts of disease-related events. In this study, we analysed information obtained from interviews with caregivers to gain insight into the onset and progression of both late-infantile and juvenile MLD, with a focus on comparisons between these two disease subtypes.

## Methods

### Patients and caregivers

Caregivers were recruited in the USA with assistance from the MLD Foundation. Equal numbers of patients with late-infantile and juvenile MLD were recruited to ensure a thorough analysis could be performed for each disease subtype. In order to be eligible for the study, caregivers had to care for, or have cared for, a living or deceased patient classified as having late-infantile or juvenile MLD in the past year (the MLD subtype was reported by the caregivers). They also had to be the primary caregiver and live in the same house as the patient; be at least 18 years of age; have access to a telephone and be available for a 60–90-min telephone interview; and be able to communicate in English. The study was approved by the Institutional Review Board (Ethical and Independent Review Services), and all caregivers signed an informed consent form before participating.

A total of 32 caregivers were interviewed. Each caregiver was questioned predominantly about one child, giving a primary sample of 32 patients with MLD. Sixteen patients had the late-infantile form of MLD and 16 had the juvenile form. During interviews, caregivers occasionally talked about siblings of the primary patient who also had MLD. Overall, information about eight siblings was collected and used in comparative analyses.

### Study design and data types

Before the interview, caregivers completed a written questionnaire, which was used to collect demographic data and core medical information (e.g. age at diagnosis). Semi-structured, in-depth interviews lasting approximately 90 min were then carried out by researchers over the telephone. All interviews were audio-recorded and transcribed.

The interviewers used discussion guides and asked caregivers open-ended questions about their experiences relating to the diagnosis, signs and symptoms and progression of MLD in their child. Interviewees were able to talk freely, providing qualitative descriptions of their experiences, such that certain topics were not discussed by all caregivers. The absence of a reported symptom or event may therefore reflect either that this was not experienced by the patient or that it was not discussed.

Information collected during interviews was used in conjunction with data collected in the pre-interview questionnaires to estimate timings of disease-related events. As these reported timings were based on retrospective recollections, they were treated as approximations.

### Data analysis

Interview transcripts were analysed using the principles of framework analysis [21], with the coding framework (Additional file 1) developed using the study objectives and previously published MLD studies [3, 4, 8]. Data were coded using qualitative data coding software (Atlas-ti, version 7.5; Scientific Software Development; Berlin, Germany) and charts were constructed using Microsoft Excel (Microsoft Corporation; Redmond, Washington, USA) to display information on the diagnostic journey and disease progression, in summarized form and/or using verbatim quotes as appropriate. Direct quotes are not reported here in order to protect the identity of patients and caregivers, given the rarity of MLD. When complete loss of a gross motor function, a fine motor or related function, or speech was described, this was defined as a ‘functional loss’. The approximate event timings provided by caregivers were used to construct individual patient timelines. When caregivers provided approximate timings within an acceptably short range (e.g. less than 1 year), the midpoint of the range was used. Charts and timelines underwent a quality review round with a second researcher before further analysis.

Charts were inspected for key themes, and comparisons were made between subgroups of interest, with a focus on patients with late-infantile versus juvenile MLD and patients who had received an HSCT compared with those who had not. Quantitative demographic data were summarized using mean and range values, unless otherwise stated.

## Results

### Patient population and demographics

A total of 32 caregivers were interviewed. All were parents of the patients, and most (30/32; 93.8%) were mothers. Sixteen patients (50.0%) were reported as having late-infantile MLD, while the remaining 16 (50.0%) had juvenile MLD (Table 1). The mean age (range) of patients with late-infantile and juvenile MLD was 7.6 (4.1–21.7) years and 20.7 (8.8–37.2) years, respectively (Table 1; the patient with late-infantile MLD aged 21.7 years had received an HSCT prior to symptom onset). Three patients (9.4%) had died prior to the interviews taking place: two with late-infantile MLD, both aged 4 years, and one with juvenile MLD, aged 19 years. HSCT (bone marrow, stem cell and/or cord blood) had been performed in 12/32 patients (37.5%; three patients with late-infantile MLD and nine with juvenile MLD).

**Table 1:** Characteristics of patients with MLD included in this analysis

Characteristic	Late-infantile MLD (*n* = 16)	Juvenile MLD (*n* = 16)
Sex, n (%)		
Male	5 (31.3)	8 (50.0)
Female	11 (68.8)	8 (50.0)
Age at interview, years^a^		
Mean (SD)	7.6 (4.6)	20.7 (8.1)
Range	4.1–21.7	8.8–37.2
Age at first symptom, years		
Mean (SD)	1.5 (0.4)	8.7 (3.6)
Range	1.0–2.4	4.0–14.8
Age at diagnosis, years		
Mean (SD)	2.6 (1.7)	11.6 (5.5)
Range	0.4–8.6	3.1–21.6
Time between first symptom and diagnosis, years^b^	(*n* = 15)	(*n* = 14)
Mean	1.2	3.7
Range	0.3–7.1	0.2–6.8
Treatments, n (%)		
Any transplant	3^c^ (18.8)	9 (56.3)
BMT	1 (6.3)	7 (43.8)
SCT	2 (12.5)	2 (12.5)
Cord blood	1 (6.3)	0 (0.0)
G-tube fitted	15 (93.8)	9 (56.3)

*BMT* bone marrow transplant, *G-tube* gastrostomy tube, *MLD* metachromatic leukodystrophy, *SCT* stem cell transplant, *SD* standard deviation

^a^For patients who had died, age at interview was defined as age at death

^b^Three patients (one with late-infantile MLD; two with juvenile MLD) were diagnosed prior to symptom onset following diagnosis of an older sibling, and therefore were not included here

^c^One patient received both BMT and SCT

### Onset of symptoms

**Fig. 1:**
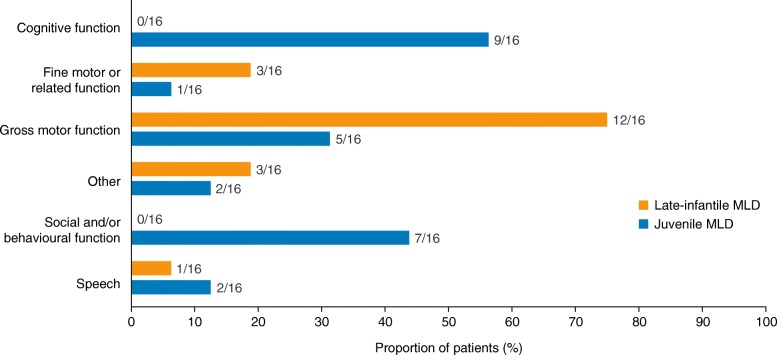
Categories of first symptoms^a^ reported in patients with late-infantile and juvenile MLD. ^a^Symptoms reported by parents were assigned to the given categories during analysis (e.g. gripping, finger movements or swallowing were classified as fine motor functions; head control, sitting and standing/walking were classified as gross motor functions). *MLD*, metachromatic leukodystrophy

Patients with late-infantile MLD had a mean age (range) of 1.5 (1.0–2.4) years at symptom onset (Table 1). The most common initial symptoms reported for these patients related to problems with gross motor function (12/16 patients; 75.0%; Fig. 1). Frequently, this was noticed as a delay in developmental progression, particularly in walking, and 11/16 patients with late-infantile MLD (68.8%) never learned to walk independently (Table 2). For example, one parent reported that their child was prone to losing his or her balance and falling over, and relied on holding hands to be able to walk.

**Table 2:** Types of symptoms^a^ or functional losses^b^ experienced by patients, either before diagnosis or at any time

	Late-infantile MLD (*n* = 16) ^c^	Juvenile MLD (*n* = 16) ^c^
Type of symptom^a^ or functional loss^b^ experienced pre-diagnosis		
Gross motor function, n (%)	15 (93.8)	9 (56.3)
Fine motor or related function, n (%)	10 (62.5)	7 (43.8)
Cognitive function, n (%)	1 (6.3)	11 (68.8)
Speech, n (%)	7 (43.8)	3 (18.8)
Social and/or behavioural function, n (%)	4 (25.0)	10 (62.5)
Other, n (%)	9 (56.3)	10 (62.5)
Types of symptom^a^ or functional loss^b^ experienced at any time		
Any gross motor function loss, n (%)	15 (93.8)	12 (75.0)
Walking impairments, n (%)		
Independent walking never developed	11 (68.8)	0 (0.0)
Walking partially lost	1 (6.3)	4 (25.0)
Walking completely lost	4 (25.0)	7 (43.8)
Any fine motor or related function loss, n (%)	15 (93.8)	12 (75.0)
Cognitive impairment, n (%)	6 (37.5)	15 (93.8)
Speech loss, n (%)	15 (93.8)	8 (50.0)
Social and/or behavioural impairment, n (%)	4 (25.0)	15 (93.8)
Seizures, n (%)	7 (43.8)	9 (56.3)
Pain (nerve or muscle spasms), n (%)	13 (81.3)	6 (37.5)
Spasticity and/or muscle spasms, n (%)	13 (81.3)	11 (68.8)
Incontinence or wearing diapers, n (%)	12 (75.0)	10 (62.5)

*MLD* metachromatic leukodystrophy

^a^Symptoms reported by parents were assigned to the given categories during analysis (e.g. gripping, finger movements or swallowing were classified as fine motor functions; head control, sitting and standing/walking were classified as gross motor functions)

^b^A functional loss was defined as a complete loss of a gross motor function, a fine motor or related function, or speech

^c^Certain symptoms may not have been queried with every parent, so a lack of report may not necessarily indicate that the child never experienced that symptom (i.e. ‘n’ may, for certain symptoms, be less than reported here)

By the time of diagnosis, all but one patient with late-infantile MLD had experienced symptoms relating to gross motor function (Table 2). Fine motor or related symptoms were also commonly reported as pre-diagnosis symptoms in the late-infantile group (10/16 patients; 62.5%). These included problems with eye movement, eating or swallowing and hand tremors. In addition, almost half of the patients with late-infantile MLD (7/16; 43.8%) experienced pre-diagnosis speech problems, with parents typically reporting a decline in ability early on in speech development. Decline in cognitive function was only reported pre-diagnosis in one patient with late-infantile MLD (6.3%).

Patients with juvenile MLD, as expected, exhibited initial symptoms later than those with late-infantile MLD, at a mean age of 8.7 (4.0–14.8) years. For these patients, first symptoms often related to changes in cognitive function (9/16 patients; 56.3%) or social/behavioural function (7/16 patients; 43.8%) (Fig. 1). Initial symptoms were usually noticed at school as a decline in academic performance, difficulty focusing or disruptive behaviour. For example, one parent explained that their child had started to make bad choices and was hitting other children. By the time of diagnosis, more than half of the patients with juvenile MLD (9/16; 56.3%) had also experienced some decline in gross motor function (Table 2), with symptoms including slowed movements, affected gait and loss of balance.

### The diagnostic process

The mean age (range) at diagnosis was 11.6 (3.1–21.6) years versus 2.6 (0.4–8.6) years for patients with juvenile and late-infantile MLD, respectively (Table 1). One patient with late-infantile MLD and two with juvenile MLD were diagnosed before symptom onset, due to previous diagnosis of a sibling.

**Fig. 2:**
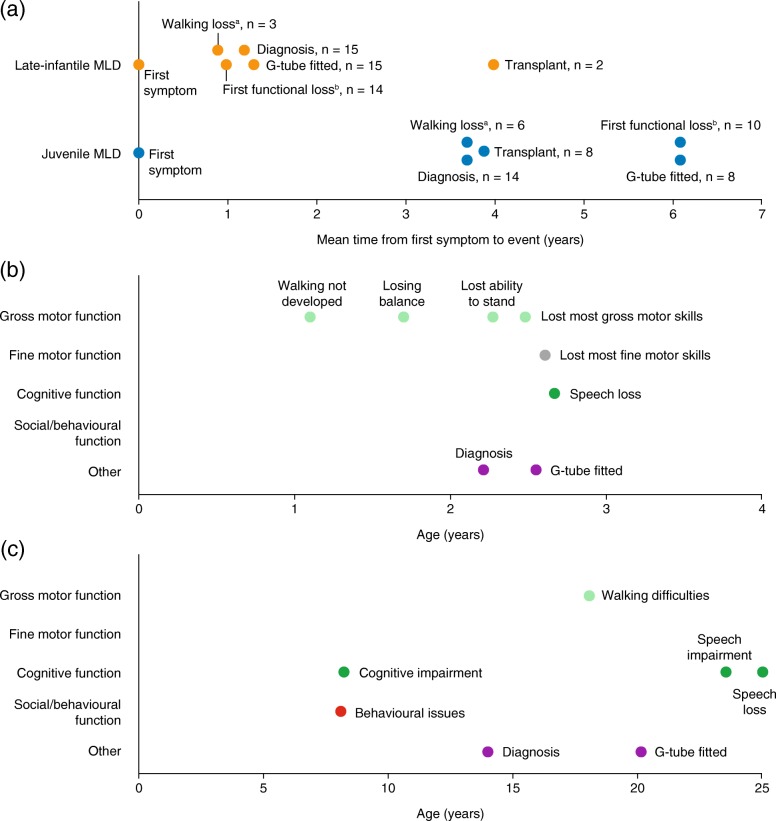
Approximate timelines of disease-related events in patients with MLD (**a**) Mean timing of events following symptom onset for patients with late-infantile MLD and juvenile MLD (**b**) Sample individual disease timeline for a patient with late-infantile MLD (**c**) Sample individual disease timeline for a patient with juvenile MLD. When parents reported an approximate timing within an acceptably short range (e.g. < 1 year), the midpoint was used. ‘n’ corresponds to the number of patients for whom information on the timing of the event was available. ^a^Walking loss was defined as a complete loss of ability to walk either assisted or unassisted. For late-infantile MLD, 11 patients who never learned to walk were not included in walking loss estimates. ^b^A functional loss was defined as a complete loss of a gross motor function, a fine motor or related function, or speech. *G-tube*, gastrostomy tube

Patients with juvenile MLD generally had a greater delay in diagnosis than those with late-infantile MLD, with a mean time from first symptom to diagnosis of 3.7 (0.2–6.8) years versus 1.2 (0.3–7.1) years (Table 1; Fig. 2a) for those who were diagnosed after symptom onset. One of the patients reported as having late-infantile MLD was not diagnosed until approximately 7 years after symptom onset. This patient also showed a delay in disease progression that was atypical of patients in the late-infantile group. For the remaining patients with late-infantile MLD, the time between symptom onset and diagnosis ranged from 4 months to 1 year and 10 months.

Initial misdiagnosis was common for patients with both subtypes of MLD. The most common incorrect diagnosis received by patients with late-infantile MLD was delayed development (5/16 patients; 31.3%), while those with the juvenile form were frequently misdiagnosed with attention deficit disorder (ADD) (7/16 patients; 43.8%). One parent reported that their child was misdiagnosed with ADD by a neuropsychologist, who had performed several tests and only identified impaired focus. Parents generally described seeing many different healthcare practitioners before receiving the correct diagnosis. Ultimately, all patients with late-infantile MLD were diagnosed by either a neurologist or geneticist, while those with juvenile MLD were diagnosed by either a neurologist (12/16 patients; 75.0%), a paediatrician (2/16; 12.5%) or an unspecified practitioner (2/16; 12.5%).

### Disease progression

Average and individual patient timelines were constructed to compare disease progression between patients with late-infantile and juvenile MLD (Fig. 2). Patients with late-infantile MLD generally experienced a rapid decline (Fig. 2a; Fig. 2b), with a mean time (range) from first symptom to first functional loss of 1.0 (0.2–4.0) years. One parent used the analogy of ‘falling off a cliff’ to describe the rapid disease progression. At the time of interview, 14/16 patients with late-infantile MLD (87.5%) had little or no functional movement remaining, or had died having lost all gross motor function. Twelve out of 16 parents (75.0%) reported that their child experienced rapid functional losses within the first 3 years of life, and four of the five patients with late-infantile MLD (80.0%) who had learned to walk later lost this ability. Most patients with late-infantile MLD also had difficulty swallowing, resulting in all but one (93.8%) having a gastrostomy tube (G-tube) fitted, at a mean time of 1.3 (0.5–4.0) years after symptom onset. While cognitive dysfunction was rarely reported as an initial symptom for late-infantile MLD, by the time of interview, 6/16 parents (37.5%) had perceived cognitive impairment in their child (Table 2). For example, one parent noticed their child ‘zoning out’ more often, while others reported that their child lost the ability to count, understand commands or retain information. However, many parents reported that their children retained the ability to recognize people (e.g. ‘perking up’ and laughing at the sound of relatives’ voices), and 9/16 patients (56.3%), aged between 4 and 21 years, were still able to smile.

The functional decline experienced by patients with juvenile MLD was generally more protracted than in patients with late-infantile MLD (Fig. 2a; Fig. 2c), with a mean time from first symptom to first functional loss of 6.1 (0.3–17.0) years. At the time of interview, all patients had displayed some level of cognitive impairment. Fifteen parents (93.8%) reported that their child had significant cognitive issues relating to information processing, reading, concentration and/or memory, which in many cases resulted in them being unable to care for themselves. For the remaining child, the lack of voluntary movement prevented communication, and therefore their level of cognitive functioning was difficult to determine. In addition, eight patients (50.0%) had little or no speech remaining by the time of interview (Table 2). Speech loss appeared to result from reduced cognitive function and/or physical impairments, although the cause was not always discernible. All but one patient with juvenile MLD (93.8%) had experienced social or behavioural problems by the time of interview, though in most cases disruptive behaviour appeared to diminish as the disease progressed. Many parents reported that their child’s social interactions were ultimately impaired by reduced motor and cognitive function, leading to loss of friendships with their peers.

Further decline in gross motor function also became common as the juvenile disease progressed. Twelve patients (75.0%) had lost a gross motor function by the time of interview; often, initial difficulties performing vigorous activities, such as running or playing sports, were followed by problems with walking and standing. While all patients with juvenile MLD learned to walk independently, 11/16 (68.8%) later partially or completely lost their walking ability, with a mean time from symptom onset to walking loss of 3.7 (0.3–10.0) years (Fig. 2a). While only 2/16 patients with juvenile MLD (12.5%) had completely lost the ability to eat, several others received a combination of solid and G-tube feeding. Nine patients (56.3%) had a G-tube fitted, at a mean time of 6.1 (0.4–12.0) years after symptom onset.

### Prevalence and outcomes of HSCT

HSCT (bone marrow, stem cell and/or cord blood) had been performed in 3/16 (18.8%) and 9/16 (56.3%) patients with late-infantile and juvenile MLD, respectively (Table 1). Most patients with late-infantile MLD were not considered for a transplant because of the advanced stage of the disease at diagnosis, while those with juvenile disease who underwent HSCT typically did so shortly after diagnosis.

Outcomes following HSCT were variable. Three patients with juvenile MLD (33.3%) were reported to have had a positive outcome, showing stabilization or improvement in some motor functions. The other six (66.7%) generally experienced a succession of functional declines, particularly immediately after transplant. Among the three patients with late-infantile MLD who underwent HSCT, one received it at the pre-symptomatic stage owing to previous diagnosis of an older sibling, and their disease did not progress as rapidly as may have been expected. Another patient received their transplant at a later stage, following symptom onset, and experienced a disease progression considered typical for late-infantile MLD. For the remaining patient, who also received their transplant after symptom onset, it was difficult to evaluate the outcome due to the relative recency of the transplant.

No patients in this study had received experimental gene therapy. One parent mentioned that they had considered this as an option, but ultimately did not proceed due to disease progression in their child.

### Sibling comparisons

Of the 32 patients in the analysis, six had siblings who had also been diagnosed with MLD (eight siblings in total; four older and four younger than the primary patient). In each of the six families, the oldest sibling was first to be diagnosed.

In all cases, the disease experience differed to some extent among siblings. In one family, two siblings with juvenile MLD both presented with cognitive impairment as their first symptom, and neither received an HSCT. The older sibling rapidly lost the ability to walk, eat and speak within 1 year from symptom onset. The younger sibling, who was diagnosed pre-symptomatically, showed a more gradual functional decline, retaining some walking ability until approximately 10 years after disease onset.

In three other families, transplant status differed among siblings. In one of these families, one sibling died shortly following their HSCT, precluding comparative analysis. One patient also died following HSCT in another family of three siblings with late-infantile MLD, but disease progression differed between the remaining two siblings. The untreated patient declined rapidly and passed away before the age of 5 years, whereas their younger sibling, who received a transplant in infancy, retained some cognitive awareness and was still alive in early adulthood. Similarly, in the remaining sibling pair, who had juvenile MLD, the patient who received an HSCT showed a delayed decline in functional skills compared with their untreated sibling.

Within the remaining two families, all siblings had juvenile MLD and all underwent HSCT at varying ages. In the first pair of siblings, transplants were performed shortly after diagnosis at approximately 6 years of age and under 1 year of age, respectively. Their parent reported that the sibling who received the earlier HSCT remained relatively asymptomatic at the time of interview, aged 6 years, while the older sibling had experienced a range of cognitive, behavioural and motor symptoms at an equivalent age. In the other family, the parent reported that earlier transplant was associated with better outcomes for their children, though the youngest child was not yet old enough to fully compare disease timelines.

## Discussion

In this study, qualitative analysis of caregiver accounts provided detailed, individual-level descriptions of disease natural history in MLD. Our findings indicate that patients with late-infantile MLD first exhibit symptoms predominantly relating to gross motor function and experience rapid functional decline, while those with juvenile MLD tend to initially develop cognitive and behavioural symptoms, followed by a more protracted disease progression. These descriptions are broadly consistent with previously published findings [2–4] and enhance our knowledge of the disease experience with MLD.

In the late-infantile group, three-quarters of parents recognized gross motor function impairments, commonly problems with independent walking, as the first disease symptoms. This is consistent with other published studies that have reported frequent falling, abnormal movements and walking difficulties in patients with this MLD subtype [3, 4, 8]. In a detailed study of motor function in a cohort of German patients, 90% of those with late-infantile MLD had shown some decline in their ability to walk or stand independently by 18 months of age, in line with the reported mean age at symptom onset observed in the present study [3]. Cognitive impairment was rarely perceived as an early symptom for patients with late-infantile MLD. This may be a consequence of the early developmental stage of these children at the onset of motor symptoms, which might make it difficult for parents to judge cognitive ability.

As expected for patients with juvenile MLD, symptom onset occurred later in childhood, and age at onset was more variable than in the late-infantile group. Although the modest patient numbers preclude a detailed subgroup comparison between those with earlier and later onset of juvenile MLD, there did not appear to be any systematic variation in the initial presentation. Cognitive and behavioural changes were the most common first symptoms, with many parents describing concentration issues or disruptive behaviour at school. Just under one-third of parents of patients with juvenile MLD also reported gross motor function impairments, such as gait disturbances, as early symptoms. These findings are also generally consistent with previous descriptions [2–4], though interestingly one study reported gait disturbances as the most common first symptom (69% of patients) [4]. The mean delay in diagnosis for patients with juvenile MLD was longer than for those with late-infantile MLD (3.7 versus 1.2 years), which may be attributable to the more specific and rapidly progressing symptoms in the late-infantile form. This delay was slightly longer than that reported in a German study [4], but considerably shorter than observed in a Brazilian cohort [22], which may reflect country-specific variation in healthcare provision for rare diseases. For both disease subtypes, initial misdiagnosis was common, and this will be an important barrier to overcome to improve early diagnosis of MLD.

We observed that patients with late-infantile MLD experienced a rapid functional decline within their first few years, which is in line with previous descriptions of this subtype [2, 3, 5]. The onset of functional loss was more delayed and variable in patients with juvenile MLD, consistent with trends observed in a detailed study of motor symptoms in German patients [3]. However, in contrast to this previous study, we found that parents of patients with juvenile MLD generally reported a delayed decline in other motor functions, such as arm movement, following walking loss. Observations relating to deteriorating speech, concentration and information processing among the juvenile group generally paralleled previous descriptions of language and cognition in MLD [4]. Interestingly, many parents implied that disruptive behavioural symptoms became less problematic as the juvenile disease progressed, which may reflect a decreased capacity for this behaviour due to declining motor and cognitive function. Although three patients in this study had died, we observed that overall, patients appeared to live longer than may be expected, with a higher mean age at interview in this study than the mean age at death reported previously (7.6 versus 4.2 years for late-infantile MLD and 20.7 versus 17.4 years for juvenile MLD) [23]. While it is important to consider the potential selection bias arising from the need for caregivers to have cared for a patient within the past year, our findings may reflect an impact of advances in supportive care on improving MLD survival rates.

Although this study focused on describing the natural history of MLD rather than evaluating therapeutic interventions, almost one-third of patients had undergone HSCT. Transplants were three times as common among patients with juvenile MLD than in those with late-infantile MLD, which is likely to reflect that this approach is generally recommended only for patients who have not experienced significant functional decline [12, 24]. Our findings suggest that in certain cases transplant may have partly stabilized or delayed disease progression. However, it is important to interpret these results with caution, due to the small sample size and the fact that it is difficult to distinguish the effects of transplant from variability in natural disease course. Larger cohort studies of patients who had received HSCTs have also revealed variable results, but have generally suggested that asymptomatic or early symptomatic patients, particularly those with juvenile MLD, had the highest chance of favourable neurocognitive and motor outcomes [11–14].

In addition to the primary patient population analysed in this study, parents also often described disease onset and progression in affected siblings. While the overall profile of symptoms experienced within sibling groups was generally similar, their timings and extent often differed. This is broadly consistent with previous reports of intrafamilial variability in the presentation of MLD, particularly among the juvenile and adult forms [1, 9, 10, 25], and suggests that additional genetic and non-genetic factors may have an important impact on disease severity and progression. In line with this, our findings also indicate that there is interindividual variability within each MLD subtype. For example, one patient classified as having late-infantile MLD experienced a delayed loss of motor function more typical of juvenile MLD. This supports the notion that although MLD is often classified into different forms based on age at onset, the distinction is likely be an over-simplification, and disease severity should perhaps be considered as a continuum [1].

Although our study provides a detailed analysis of the disease experience in MLD, it is also important to recognize potential limitations of the data. The sample size, while reasonable for a rare disease, was relatively small, and all patients were from the USA. It is therefore not clear to what extent these findings may apply to the global population of patients with MLD. In addition, the interview methodology used has inherent limitations due to the need for caregivers to retrospectively remember experiences and timings of events. This may have resulted in potential recall bias and data inaccuracies, particularly given that the reports were not cross-checked against medical records, and timings should therefore be considered as approximations only. There was also some variability in the data available for each participant, which arose from the open-ended nature of the interviews. Finally, the fact that some individuals had received an HSCT made it more challenging to define the natural history of the disease. Despite these limitations, the findings were largely consistent with previous reports, and make an important contribution to our understanding of MLD.

## Conclusions

These results highlight the value of caregiver interviews as a useful methodology for obtaining detailed insights into rare diseases such as MLD. In line with previous reports, we found that late-infantile MLD is generally characterized by early motor symptoms and rapid functional decline, while patients with juvenile MLD commonly experience initial behavioural and cognitive symptoms followed by a more delayed disease course. Further research is needed to fully understand the influence of genetic and environmental factors on disease phenotype and interindividual variability, as well as the impact of interventions such as HSCT. Overall, these findings make an important contribution to our understanding of the experiences of patients with MLD and will provide a valuable starting point for assessing the effects of disease-related interventions in the future.

## Additional file

Additional file 1: Final coding framework used in the qualitative analysis. A table showing the coding framework used during qualitative analysis of the interview transcripts; containing sections on the diagnostic journey and disease progression. (PDF 59 kb)

## Abbreviations

ADD: Attention deficit disorder
ASA or ARSA: Arylsulfatase A
G-tube: Gastrostomy tube
HSCT: Haematopoietic stem cell transplant
MLD: Metachromatic leukodystrophy
