# Supplementary material for: Insights into the natural history of metachromatic leukodystrophy from interviews with caregivers
Source: Orphanet J Rare Dis. 2019 Apr 29;14:89. doi: 10.1186/s13023-019-1060-2 (PMC6489348; doi:10.1186/s13023-019-1060-2)
Supplement: Supplementary file 1 — Final coding framework used in the qualitative analysis. A table showing the coding framework used during qualitative analysis of the interview transcripts; containing sections on the diagnostic journey and disease progression. (PDF 59 kb) [file 13023_2019_1060_MOESM1_ESM.pdf]

Additional file 1: Final coding framework used in the qualitative analysis
Harrington *et. al*

Diagnostic journey (prediagnosis)

Code number	Code label	Code description
**Diagnosis process**		
02-01-01	Age at diagnosis	Age of child when he or she received MLD diagnosis
02-01-02	Time to diagnosis	Length of time between first seeking medical help or advice to final MLD diagnosis
02-01-03	Misdiagnosis	Misdiagnoses received from HCPs before MLD diagnosis
02-01-04	HCPs seen prediagnosis	HCPs seen leading up to diagnosis
02-01-05	HCPs seen at diagnosis	HCP who made MLD diagnosis
02-01-06	Services used	Tests or procedures undergone, treatments received and services used (e.g. hospitalization) leading up to diagnosis
**Symptoms**		
02-02-01	Initial symptom	First symptom, or first combination of symptoms, that occurred or were noticed before diagnosis
02-02-02	Subsequent symptoms	Other symptoms that occurred or were noticed after the first symptoms before diagnosis
02-02-03	Red flag	Key symptom or event that alerted the caregiver that something was wrong or that prompted them to seek medical advice or help
**Financial impact**		
02-03-01	Costs	Nature and impact of any costs incurred leading up to diagnosis

HCP, healthcare professional; MLD, metachromatic leukodystrophy

Disease progression

Code number	Code label	Code description
**Gross motor symptoms**		
03-01-01	Head control	Able to support and control own head
03-01-02	Rolling	Rolling over from front to back or back to front
03-01-03	Sitting	Holding a sitting position
03-01-04	Crawling	Crawling on hands and knees

03-01-05	Standing	Pulling self to a standing position; bearing own weight on legs
03-01-06	Cruising	Shuffling along furniture
03-01-07	Walking	Walking, including going up and down stairs or steps
03-01-09	Beyond walking	Running, playing sports, riding a bike
03-01-10	Arm movements	Moving, lifting or using arms
03-01-08	General gross motor symptoms	Gross motor symptoms that are not specified in detail
**Fine motor and related symptoms**		
03-02-01	Gripping, grasping, holding	Holding an object in the hand (e.g. holding a toy, holding hands); closing fingers around an object
03-02-02	Finger and hand movements	Picking up or moving objects with fingers (e.g. picking an object up, using buttons); moving fingers and hands
03-02-03	Writing	Drawing, colouring, writing or scribbling with a pen or pencil
03-02-04	Swallowing, eating	Being able to open mouth, chew and swallow food
03-02-05	Smiling	Using mouth to smile and laugh
03-02-06	Eye movement	Tracking an object or person from left to right or up and down; intentionally opening and closing eyelids
03-02-07	Involuntary movement	Tremor, twitching, shaking
03-02-08	General fine motor or related symptoms	Fine motor symptoms that are not specified in detail
**Cognitive symptoms**		
03-03-01	Speech	Babbling, talking
03-03-02	Gesturing	A bodily action (e.g. moving hand, head, arms, etc.) intended to communicate
03-03-03	Reading	Reading written language
03-03-04	Information processing	Understanding and processing information: following instructions, completing tasks, multitasking, recognizing people, awareness of surroundings, academic performance

03-03-05	Memory	Remembering and recalling facts and/or past events
03-03-06	Concentration	Paying attention, focusing on a task
03-03-08	Communication	Purposefully communicating wants, preferences, choices, or needs through movement or other non-verbal means
03-03-07	General cognitive symptoms	Cognitive symptoms that are not specified in detail
**Social, emotional and/or behavioural symptoms**		
03-04-01	Interacting or playing with others	Reciprocal play, taking turns, initiating and maintaining conversation
03-04-02	Social engagement	Reacting and responding, positively seeking social interaction, withdrawing
03-04-03	Emotions	Bad temper, moods, aggression, sadness
03-04-04	Incongruent emotions	Inappropriate, unusual or excessive expressions of emotion (e.g. crying, smiling, laughing)
03-04-05	Socially inappropriate behaviour	Behaviours that are socially undesirable or inappropriate (e.g. lack of inhibition, biting)
03-04-07	Affective symptoms	Anxiety, depression or psychotic problems (e.g. hallucinations)
03-04-06	General social, emotional or behavioural symptoms	Behaviours that are social, emotional or behavioural in nature but are not specified in detail
**Other symptoms**		
03-05-06	Muscle symptoms	Spasms, spasticity, tightness, hypertonia, hypotonia, cramps
03-05-04	Pain	Pain or discomfort
03-05-02	Seizures	Seizures
03-05-03	Toileting	Bladder and bowel control, bowel movements or constipation
03-05-07	Self-care functioning	Being able to dress, wash and feed self
03-05-05	Vision and hearing	Being able to see and hear
03-05-01	Other symptoms	Any other symptoms not included in coded themes

MLD, metachromatic leukodystrophy
